# Antidiuretic hormone deficiency secondary to inactive hydrocephalus: a case report

**DOI:** 10.1186/s13256-024-04467-6

**Published:** 2024-03-31

**Authors:** Anuradha Kadel, Nikita Kharal, Srijana Sapkota, Prakash Pokhrel, Arun Kumar Sharma, Aseem Bhattarai, Mithileshwer Raut, Raju Kumar Dubey, Eans Tara Tuladhar, Vijay Kumar Sharma, Apeksha Niraula

**Affiliations:** 1 Department of Clinical Biochemistry, Institute of Medicine, Maharajgunj Medical Campus, Kathmandu, Nepal; 2 Department of Paediatrics, Institute of Medicine, Maharajgunj Medical Campus, Kathmandu, Nepal

**Keywords:** Central diabetes insipidus, Inactive hydrocephalus, Hypernatremia, Pediatrics, Arginine vasopressin

## Abstract

**Background:**

Diabetes insipidus is a syndrome characterized by polyuria, which is almost always associated with polydipsia. The most frequent cause is central diabetes insipidus, which is the result of an inadequate secretion of the antidiuretic hormone, and diagnosis involves differentiating it from other causes of polyuria and polydipsia.

**Case presentation:**

Here, we present a clinical case of a previously healthy 13-year-old Nepali boy, who, in December 2022, was found to have intense polydipsia accompanied by polyuria. He had bilateral lower limb weakness at the time of presentation. Biochemical evaluation demonstrated raised serum sodium (181 mEq/L), serum creatinine (78 μmol/L), and serum uric acid (560 μmol/L) with suppressed serum potassium (2.7 mEq/L), which was the major concern to the clinicians. Further laboratory workup revealed an increased serum osmolarity (393.6 mOsm/kg) with reduced urine osmolarity (222.7 mOsm/kg). On contrast magnetic resonance imaging of the brain, a thick-walled third ventricular cyst with bilateral foramen obstruction, thin membrane-like structure at top of aqueduct of Sylvius with gross obstructive hydrocephalus (inactive), and compressed and thinned pituitary gland with no bright spot was observed. The laboratory findings, radiological findings, and case presentation provided the provisional diagnosis of diabetes insipidus due to hydrocephalus and third ventricular cyst.

**Conclusions:**

Central diabetes insipidus due to hydrocephalus, though rare, can have serious complications including the predilection to develop a deficit of other pituitary hormones. Thus, even if hydrocephalus is dormant with normal intracranial pressure, it must be addressed during investigations of central diabetes insipidus.

## Background

Central diabetes insipidus (CDI) implies a serious underlying disease in children [1]. Polyuria and polydipsia are the hallmarks of CDI, which is brought on by a lack of arginine vasopressin (AVP), an antidiuretic hormone that stimulates the reabsorption of free water by acting on V2 receptors in the kidney [2]. Important and well-described causes of childhood CDI include tumors and malformations of the central nervous system (CNS), histiocytosis, and hypothalamic–pituitary region anomalies by surgery, radiation, and trauma [3–5]. However, a rare and less recognized cause of CDI includes hydrocephalus. Most of the cases due to hydrocephalus used to be categorized as idiopathic CDI but the development of laboratory examinations and imaging technologies have brought advancement in the diagnosis and etiological identification of CDI.

However, only a few CDI cases due to hydrocephalus have been documented in South East Asia and even worldwide in medical journals. Here, we present a case of diabetes insipidus (DI) presenting polyuria, polydipsia, and bilateral lower limb weakness as the presentation with the clinical features of panhypopituitarism. After a thorough investigation ruling out the differential diagnosis, based on laboratory and radiological findings a final diagnosis of CDI secondary to inactive hydrocephalus was made.

This is one of the few cases of CDI with hydrocephalus as the central etiology. This case allows for speculation of predisposing risk factors of this phenomenon and can aid in early management of CDI and possibly associated panhypopituitarism.

## Case presentation

A 13-year-old Hindu school-going Nepali boy presented to our emergency room with acute onset of bilateral lower extremity weakness and unsteady gait. The patient was in his normal state of health until 5 days before, but recently he complained about weakness of the lower limb that was insidious in onset. The symptoms initially started as an inability to stand properly with pain in a limb while standing. The symptoms gradually progressed and the child was completely bedridden, which prompted a visit to our emergency department from where he was shifted to the pediatric high-dependency unit (HDU). Past history revealed an excessive intake of water with the inability to hold urine during day and night time for the last 4 months but no history of urgency, burning micturition, fever, or passage of red-colored urine. The patient was taking no medications. He did not have any other constitutional symptoms, including fever, rash, myalgias, diarrhea, or seizures. There was no significant family history of any chronic illness. Past medical history of the patient revealed no tuberculosis, recent vaccination, meningitis, or head trauma. However, he had a history of weakness of the lower limb since birth in the form of limping gait. He was not able to walk until the third year of life, and there was halt in his growth (height and weight) since he was 6 years old.

On the day of admission to our hospital, a physical examination revealed a temperature of 98 °F, a heart rate of 94 beats per minute, a respiratory rate of 32 breaths per minute, SpO_2_ of 98%, and a blood pressure of 100/60 mmHg. Though he was a healthy term infant at birth, his current height and weight were 115 cm and 20 kg, respectively. Further analysis revealed his weight-to-age ratio, height-to-age ratio, and body mass index all to be < 3rd percentile of the age-matched normal reference range. He had not developed secondary sexual characteristics. Examination revealed small soft testes and penis, no male body hair, hydrocephalic head configuration, and ataxic gait. Physical examination was otherwise unremarkable. Neurological examination showed child to be drowsy with a Glasgow coma scale (GCS) score of 12/15, had brisk knee jerk reflex with downward plantar reflex, bilateral lower limb strength of 1/5, and 2+ pupillary light reflex. No signs of meningeal irritation and cerebellar sign were seen. Sensory examination was unremarkable. There was polyuria with urine output of 9 ml/kg/hour and specific gravity of 1.010.

A complete blood count revealed a hemoglobin concentration of 12.8 g/dL, hematocrit of 39.7%, a mean corpuscular volume of 66.50 fL, and a white cell count of 11,100/cumm (normal range, 4000–11,000/cumm) with 53% neutrophils, 37% lymphocytes, and 8% monocytes. Further investigation showed a high serum sodium of 181 mEq/L, rising to a maximum of 190 mEq/L (normal range, 135–146 mEq/L) at 2 days following admission. Potassium, which was initially toward the lower range (2.7 mEq/L) (normal range, 3.5–5.2 mEq/L) during the time of admission was brought to normal after the correction by normal saline with potassium chloride (KCl) within 1 day of admission (4.0 mEq/L). Later, N/2 normal saline with 5% dextrose was also added. Urea, alanine aminotransferase (ALT), and aspartate aminotransferase (AST) were normal as were serum protein and calcium levels. Random glucose was 7.9 mmol/L (normal, 3.8–7.8 mmol/L) with increased serum creatinine and uric acid up to 78 μmol/L (normal, 23–68 μmol/L) and 560 μmol/L (normal, 208–428 μmol/L), respectively.

**Fig. 1:**
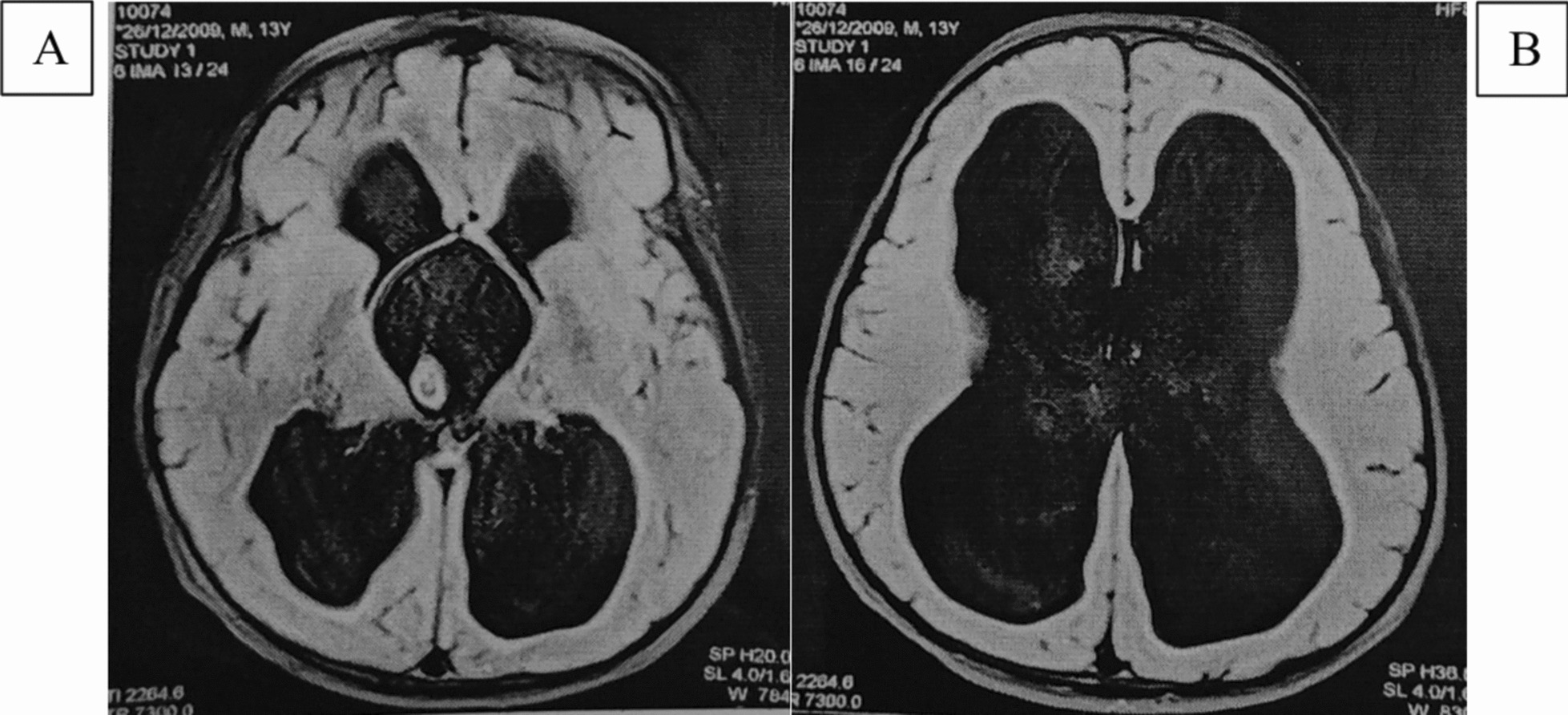
Axial FLAIR MRI of brain showing (**A**) dilated third and lateral ventricle, (**B**) dilated lateral ventricle

**Fig. 2:**
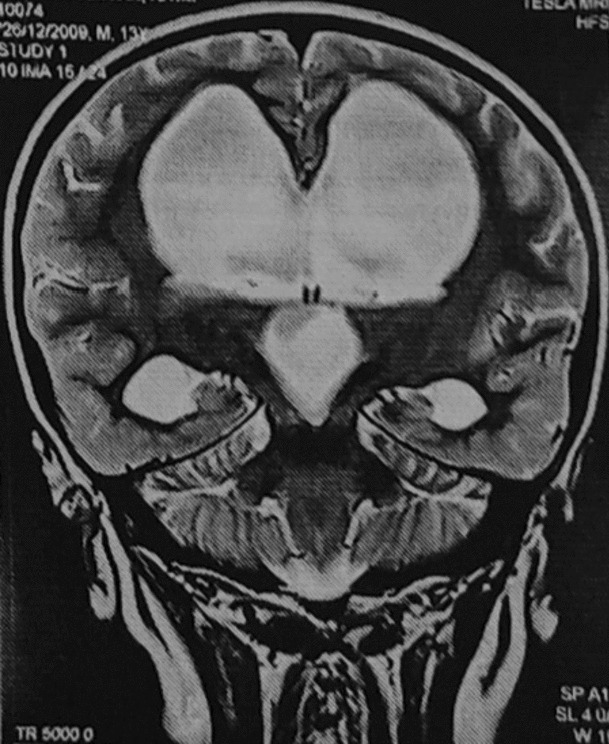
T2-weighted coronal MRI of brain showing dilated lateral and third ventricle

On the second day of admission, the child was transferred to pediatric intensive care unit (ICU) for falling GCS (from 12/15 in the morning to 8/15 in the evening) and severe hypernatremia (Na^+^ 180 mEq/L). A microbiological investigation of urine was sent and showed a white cell count of 1–2 cells/high-power field (HPF), a trace amount of albumin, and a 0–1 granular cast/HPF. Serum and urine osmolarity tests were performed to identify the cause of polyuria and polydipsia, which showed increased serum osmolarity (393.6 mOsm/kg) (normal, 285–295 mOsm/kg) with decreased urine osmolarity (222.7 mOsm/kg) (normal, 500–800 mOsm/kg). Psychogenic polydipsia (PPD) was ruled out in the instance of elevated serum sodium level and osmolarity. To support this, contrast magnetic resonance imaging (MRI) of the brain was performed (Figs. 1 and 2), which showed a thick-walled third ventricular cyst with bilateral foramen obstruction, the thin membrane-like structure at top of aqueduct of Sylvius with gross obstructive hydrocephalus (inactive), and compressed and thinned pituitary gland with no bright spot suggesting a diagnosis of CDI. We were not able to perform a water deprivation test. Pansinusitis and bilateral otomastoiditis were also observed on MRI. In children, the aforementioned condition is more likely to be associated with clinical symptoms and endocrinopathies, particularly growth hormone deficiency, hypogonadotropism, or multiple pituitary hormone deficiencies [6]. To further assess pituitary function, endocrine examinations were requested that showed free thyroxine (fT4) and free tri-iodothyronine (fT3) levels to be decreased with elevated prolactin level, with values of 7.31 pmol//L (normal, 9.0–19.0 pmol//L), 1.99 pmol/L (normal, 2.4–6.0 pmol//L), and 76.3 ng/mL (normal, 3.4–19.4 ng/mL), respectively. Thyroid stimulating hormone (TSH) (2.50 μIU/mL) (Normal, 0.35–4.94 µIU/ml), serum cortisol (14.6 μg/dL) (Normal, Morning: 3.7–19.4 µg/dl), luteinizing hormone (LH) (3.03 μIU/mL)(Normal, 1.1–8.2 µIU/ml), and follicle stimulating hormone (FSH) (2.81 μIU/mL) (Normal, 1.5–11.5 µIU/ml) were within normal range. A diagnosis was made of CDI secondary to the third ventricular cyst with inactive hydrocephalus based on MRI findings.

Treatment with a desmopressin tablet (0.05 mg) resulted in the resolution of his polydipsia and polyuria and the serum electrolytes were restored to normal. Endoscopic fenestration with endoscopic third ventriculostomy (ETV) was done to remove the cyst and treat obstructive hydrocephalus. A biopsy was taken from the cyst wall, which was found to be normal. The patient recovered from symptoms of polyuria and polydipsia with desmopressin therapy, until the tenth month of discharge, when he was presented to pediatric emergency room again with the complain of seizure and polyuria, which was later diagnosed to be due to CDI-induced hypernatremia and obstructive hydrocephalus. The patient was stabilized by desmopressin nasal spray and injectable Levera for seizure, which was later changed to oral. No intervention was needed for obstructive hydrocephalus on discussion with the neurosurgical team.

## Discussion and conclusion

CDI is a clinical syndrome of thirst and polyuria due to insufficient vasopressin release into the circulation despite an appropriate osmotic stimulation. AVP regulates water balance by promoting free water reabsorption via the V2 receptor in the kidney. In physiological settings, plasma osmolality (or serum Na^+^) is the primary regulator of AVP release and synthesis [7]. The osmoregulation of the AVP neuron system is so precise that merely a 1–2% changes in blood Na^+^ levels considerably promote its release as well as AVP gene transcription [8].

In one-third to a half of cases, CDI is idiopathic; it is often sporadic but may be autosomal dominant [9]. Past work has suggested an autoimmune etiology in some cases of the idiopathic group [10]. A major review of the etiology of CDI in 92 cases identified no cases of hydrocephalus [11]. Another review of CDI cases with childhood presentation identified intracranial defects in 14% of cases [3]. In this series, hydrocephalus is indicated as a rare cause, but the insufficient incidence is provided. The shifting distribution of CDI etiologies together with a declining prevalence of the idiopathic category is consistent with the development of our diagnostic skills. Our case highlights the unique etiology of CDI due to inactive hydrocephalus, which can also add value to the medical literature.

Diagnosis of CDI involves the presence of hypotonic polyuria on 24 hour urine volume, with the exclusion of diabetes mellitus, renal impairment, hyperglycemia, hypercalcemia, and hypokalemia by baseline laboratory tests [12]. The fact that our patient showed initial hypokalemia shifted our diagnostic pathway initially. However, plasma sodium concentration, serum, and urine osmolality gave some useful clues to the differential diagnosis. Severe hypernatremia and high serum osmolality with decreased urine osmolality were the key fact in our case, which brought CDI in our provisional diagnosis. The water deprivation test could have further attributed to the final diagnosis; however, we could not perform this test. In individuals with polyuria and polydipsia, the water deprivation test is the gold standard in distinguishing CDI or nephrogenic DI from PPD. Additionally, as the second step of the water deprivation test, administration of an AVP analog (desmopressin acetate) may aid in differentiation of central from nephrogenic DI, as the response to an antidiuretic is theoretically preserved in central DI but not in nephrogenic DI [13]. Because of the inability to perform the water deprivation test, in our case, PPD was ruled out with high serum sodium and osmolarity while a diagnosis of CDI was assisted by absence of bright spot and resolution of polyuria, polydipsia, and electrolyte abnormalities on treatment with desmopressin.

A compressed thinned pituitary gland as a result of extension of the third ventricle to sella and obstructive hydrocephalus could be responsible for pituitary hypofunction. In our case, the patient was between the ages of 9 and 14 years, which is considered the pubertal period for males. The clinical features of patient such as short stature, delayed puberty, lack of proper development suggested by < 3rd percentile value of body mass index (BMI) compared with the age- and pubertal status-matched normal reference range could be the result of atrophy of pituitary gland. Growth hormone could have been assayed to identify the cause of inadequate growth in the patient. FSH and LH levels are low in the body before puberty. Approximately 1 year prior the onset of puberty, CNS inhibition of GnRH lessens, leading to a disproportionate rise in the release of FSH and LH with greater amplitude of LH peaks [14]. Depressed pituitary gland along with hyperprolactinemia and clinical features suggest the possibility of hypogonadism in the patient; however, LH and FSH was found to be normal. Testosterone could have aided in the confirmation; however, we had no data regarding the test. Thus, a potential corticotropic deficit, a probable gonadotropic deficit, and a thyrotropic deficit (secondary hypothyroidism) with low T4 and normal TSH visualize the presentation of possible panhypopituitarism in the patient.

The recommended screening for complete pituitary insufficiency usually includes morning cortisol concentrations, fT4, estradiol in women and testosterone in men, insulin-like growth factor (IFG)-1, and prolactin. If abnormalities are found, additional measurements include TSH for the thyrotropic axis, FSH and LH for the gonadotropic axis, a stimulation test [insulin-induced hypoglycemia test or growth hormone releasing hormone (GHRH)-L-arginine test] for assessment of the somatotropic axis and adrenocorticotropic hormone (ACTH) stimulation test for corticotropic axis [15].

We relied on radiological findings for the final diagnosis. The absence of bright spots, a third ventricular cyst with hydrocephalus, and a depressed pituitary gland confirmed the etiology of CDI.

The etiology of our case was similar to a 48-year-old female in the UK as described by Menzies *et al*. [12] in which late presentation of CDI secondary to longstanding hydrocephalus with impaired hypothalamic–pituitary function was observed [16]. Additionally, Borenstein-Levi *et al*. described two cases of transient DI in neonates with posthemorrhagic hydrocephalus showing that such DI could be reversible with the reduction of ventricular size (either because of spontaneous resolution or the placement of the V–P shunt), which is similar to our study [17]. Another study by Taieb *et al*. showed the reason for atrophy of pituitary gland and thus pituitary hormones deficiency to be increased intracranial hypertension [18].

With emerging advances in technologies, it can be presumed that several occasions classified as idiopathic CDI in the absence of CT scans would actually have been the result of intracranial abnormalities. A lesion of the hypothalamic paraventricular nuclei or neurohypophysis is seen in CDI. We can hypothesize that hydrocephalus can cause CDI by compressing the aforementioned structures and dilatation of the third ventricle. This might also lead to the deficiency of other pituitary hormones.

Children and young adults with CDI, especially those with dilated third ventricle, hydrocephalus, and a reduction in the pituitary gland size, are prone to developing a deficiency of other pituitary hormones as well [5]. CDI due to hydrocephalus, though rare, can lead to adverse outcomes. Thus, even if hydrocephalus is inactive with normal intracranial pressure, it must be addressed while investigating the etiology of CDI and other pituitary hormone deficiencies for appropriate and meticulous patient care.

## Data Availability

All the required data are available in the manuscript.

## Abbreviations

CDI: Central diabetes insipidus
AVP: Arginine vasopressin
HDU: High-dependency unit
CT: Computed tomography
MRI: Magnetic resonance imaging
GCS: Glasgow coma scale
ICU: Intensive care unit
ETV: Endoscopic third ventriculostomy
TUTH: Tribhuvan University Teaching Hospital
